# 

**Published:** 1906-06-23

**Authors:** 


### Medical Appointments and Vacancies.

### VACANCIES.

Belgrave Hospital for Children, Clapham Road, S.W.—Assistant Physician.

Cardiff Asylum.—Medical Superintendent.

Cardiff, University College of South Wales and Monmouthshire.—Lecturer on Materia Medica and Pharmacology.

East London Hospital for Children and Dispensary for Women, Shadwell, E.—House Physician.

Exminster, Devon County Asylum.—Assistant Medical Officer.

Fulbourn, near Cambridge, Lunatic Asylum.—Senior Assistant Medical Officer.

Gloucester General Infirmary and Eye Institution.—Assistant House Surgeoncy.

Gravelly Hill, near Birmingham, Jaffray Branch of the General Hospital.—Resident Medical and Surgical Officer.

Greenock Infirmary.—Assistant House Surgeon.

Hospital for Sick Children, Great Ormond Street, London, W.C.—House Surgeon.

Indian Medical Service.—Examination for not less than Twenty Commissions.

Leicester Infirmary.—Refraction Assistant to the Ophthalmic Department.

London (Royal Free Hospital) School of Medicine for Women, 8 Hunter Street, W.C.—Second Demonstrator of Anatomy.

New Hospital for Women, Euston Road, N.W.—Two House Surgeons (females). Also Clinical Assistant.

Poplar Hospital for Accidents, Poplar, E.—Senior House Surgeon.

Royal Veterinary College, Camden Town.—Professor of Biology. Also Lecturer on Physics.

Royal Waterloo Hospital for Children and Women, Waterloo Road, S.E.—Honorary Dental Surgeon.

St. Bartholomew's Hospital and College.—Demonstrator of Chemistry. Also Demonstrator of Physics.

Sheffield Children's Hospital.—Honorary Medical Officer.

Stafford, Staffordshire General Infirmary.—Assistant House Surgeon.

Wadsley, near Sheffield, West Riding Asylum.—Fifth Assistant Medical Officer.

Worcester General Infirmary.—House Physician.

### Radcliffe Infirmary, Oxford.

Captain G. C. Rynd, who has been Acting-Secretary at the Radcliffe Infirmary, Oxford, since the death of A. C. Virgo, has been appointed Secretary.

### The Nurses' Bookshelf

### A HANDBOOK FOR NURSES.

By J. K. WATSON, M.D.Edin., M.B., C.M. **5/- net; 5/4** post free.

THIRD EDITION. **With Examination Questions based on the contents of the Chapters.**

The most complete and up-to-date Nursing Handbook. The Author's purpose has been to supply in one volume that information which nurses have been compelled hitherto to extract from various medical works, and to present that information in a suitable form.

### A COMPLETE HANDBOOK OF MIDWIFERY.

By J. K. WATSON, M.D.Edin., M.B., C.M. **6/- net; 6/4** post free.

**Profusely Illustrated with 143 Diagrams.**

This Handbook has been designed to meet the requirements of the **CENTRAL MIDWIVES BOARD,** and is practically indispensable to those nurses who desire to present themselves for the Board's examination.

### THE NURSING OF INFECTIOUS DISEASES.

By F. J. WOOLACOTT, M.A., M.D. **2/6** net; **2/9** post free.

This work contains a fairly complete description of the chief infectious diseases of this country, and of the measures, both preventive and curative, usually taken in dealing with them. No nurses who desire to be fully qualified can afford to be without this most useful book.

### SURGICAL INSTRUMENTS AND APPLIANCES USED IN OPERATIONS.

**An Illustrated and Classified List, with Explanatory Notes.**

By HAROLD BURROWS, M.B.Lond. **1/6** net; **1/8** post free.

Contents: Instruments required in Special Operations—The Anæsthetist's Table—Out-patients' Department and Consulting Rooms—Preparation for Operation in a Private House, &c., &c.

### LECTURES ON HOME NURSING FOR THE POOR.

By A DISTRICT NURSE. **1/6** net; **1/8** post free.

Contents: General Care of the Sick Person and the Sick Room—The Doctor—Prevention and Treatment of Bedsores—Poultices—Fomentations—Dressing of Wounds—Dangers of Quick Healing and Quacks—Typhoid: What It is, and How to Nurse It—Infants and Children—Convalescence—Emergencies.

### THE CARE OF CHILDREN:

**Practical Hints for Mothers and Nurses at Home and Abroad.**

By ROBT. J. BLACKHAM, D.Ph (Lond.). **1/6** net; **1/8** post free.

Contains: Hints on Food, Drink and Medicines, Clothing, Cleanliness, Rest and Exercise, Infectious Diseases, on the Nursery, on Nursery "First Aid," on Nursery Cooking, &c., &c.

***Complete Catalogue sent post free on application.***

### THE SCIENTIFIC PRESS, LTD.

**Nursing Publishers and Booksellers,**

**28 & 29 SOUTHAMPTON STREET, STRAND, LONDON, W.C.**

**Publications.**

### NEURASTHENIA, Clinical Lectures on.
By THOMAS D. SAVILL, M.D. Lond. 2nd Edition.

*Physician to the Hospital for Diseases of the Nervous System, &c. &c.*

"The author has had exceptional opportunities for study in this speciality."—THE CANADIAN JOURNAL. "Goes very fully into the details of treatment."—THE LANCET.

H. J. GLAISHER, 57 WIGMORE STREET, W. **5s. net; 5s. 4d. by post.**

### DISEASES OF THE ANUS & RECTUM
By D. H. GOODSALL, F.R.C.S., and W. ERNEST MILES, F.R.C.S.,

Senior Surgeon Metropolitan Hospital; late Senior Surgeon, St. Mark's Hospital. Surgeon Cancer Hospital; Surgeon Gordon Hospital.

PART I.—Containing Chapters on Anatomy, General Diagnosis, Abscess, Ano-Rectal Fistula, Recto-Urethral, Recto-Vaginal and Recto-Vesical Fistula, Sinus over the Sacrum and Coccyx, Anal Fissure and Hæmorrhoids (or Piles). With 91 Illustrations, 8vo. 7s. 6d. net.

PART II.—Containing Chapters on Prolapse, Invagination Ulceration, Stricture, Malignant Growths, Benign Tumours, Foreign Bodies, Pruritis Ani, and Syphilis. With 44 Illustrations, 8vo. 6s. net.

LONGMANS, GREEN & CO., London, New York, Bombay.

### WORKS BY DAVID WALSH, M.D.
**RÖNTGEN RAYS IN MEDICAL WORK.** *Third Edition.* 12s. 6d.
**THE HAIR AND ITS DISEASES.** 2s. 6d. net. A Standard Monograph. Baillère, Tindall, & Cox, London.
**GOLDEN RULES OF SKIN PRACTICE.** 1s. net. *Second Edition.* Wright & Co., Bristol.
**AGE AND OLD AGE: Handbook.** 2s 6d. R. A. Everett & Co., London.

JUST PUBLISHED. Price **2s. 6d.** net.

### CARCINOMA OF THE RECTUM.
By F. SWINFORD EDWARDS, F.R.C.S.,

Surgeon to the West London Hospital; Senior Surgeon to St. Peter's Hospital for Stone, and to St. Mark's Hospital for Fistula, &c.

London: BAILLIÈRE, TINDALL, & COX, 8 Henrietta Street, Covent Garden.

THE MOST EFFECTUAL BANDAGE FOR THE CURE OF ULCERS AND OTHER DISEASES OF THE LEG.

Recently Published, Demy 8vo. cloth, 7s. 6d.

### THE INFLAMMATION IDEA IN GENERAL PATHOLOGY.
**By W. H. RANSOM, M.D., F.R.S., &c.**

"The Essay has more than weight enough to recommend itself to the consideration of skilled inquirers specially interested in its problems, for it bears on every page evidences of original observation and independent thought."—*Scotsman.*

WILLIAMS & NORGATE, 14 Henrietta St., Covent Garden, London, W.C.

